# Icariside II, a natural mTOR inhibitor, disrupts aberrant energy homeostasis via suppressing mTORC1-4E-BP1 axis in sarcoma cells

**DOI:** 10.18632/oncotarget.8538

**Published:** 2016-04-01

**Authors:** Chao Zhang, Lei Yang, Ya-di Geng, Fa-liang An, Yuan-zheng Xia, Chao Guo, Jian-guang Luo, Lu-yong Zhang, Qing-long Guo, Ling-yi Kong

**Affiliations:** ^1^ State Key Laboratory of Natural Medicines, Department of Natural Medicinal Chemistry, China Pharmaceutical University, Nanjing, China

**Keywords:** Icariside II, aberrant energy homeostasis, glycolysis, mRNA translation, mTORC1-4E-BP1 axis

## Abstract

The aberrant energy homeostasis that characterized by high rate of energy production (glycolysis) and energy consumption (mRNA translation) is associated with the development of cancer. As mammalian target of rapamycin (mTOR) is a critical regulator of aberrant energy homeostasis, it is an attractive target for anti-tumor intervention. The flavonoid compound Icariside II (IS) is a natural mTOR inhibitor derived from *Epimedium. Koreanum*. Herein, we evaluate the effect of IS on aberrant energy homeostasis. The reduction of glycolysis and mRNA translation in U2OS (osteosarcoma), S180 (fibrosarcoma) and SW1535 (chondrosarcoma) cells observed in our study, indicate that, IS inhibits aberrant energy homeostasis. This inhibition is found to be due to suppression of mammalian target of rapamycin complex 1 (mTORC1)-eukaryotic translation initiation factor 4E-binding protein 1 (4E-BP1) axis through blocking the assembly of mTORC1. Furthermore, IS inhibits the cap-dependent translation of c-myc through mTORC1-4E-BP1 axis which links the relationship between mRNA translation and glycolysis. Inhibition of aberrant energy homeostasis by IS, contributes to its *in vitro* and *in vivo* anti-proliferation activity. These data indicate that IS disrupts aberrant energy homeostasis of sarcoma cells through suppression of mTORC1-4E-BP1 axis, providing a novel mechanism of IS to inhibit cell proliferation in sarcoma cells.

## INTRODUCTION

Sarcomas are mesenchymal malignancies that exhibit a higher percentage of overall cancer morbidity and mortality in children and adolescents than in adults [1]. Based on a primary location, sarcomas can be grouped into primary skeletal sarcoma (osteosarcoma and chondrosarcoma) and soft tissue sarcoma (fibrosarcoma) [2]. Current treatment of sarcoma is surgery and combinational chemotherapy that includes methotrexate, cisplatin, doxorubicin, ifosfamide, etoposide and 5-fluorouracil. However, the survival rate for patients with sarcoma has remained unchanged since the introduction of chemotherapeutics in the 1970s [3]. Therefore, identification if a new therapeutic strategy is required. Recently, great advances have been made in the understanding of the molecular biology of sarcoma [4, 5]. The deeper understanding of some relevant pathways and their key effectors has permitted the development of new drugs able to intervene with sarcoma development.

Aberrant energy homeostasis is a key factor in metabolic disorders such as cancer. mRNA translation, consumes most energy in cancer cells [6, 7] that positively correlated with proliferation rates [8]. Therefore, upregulated mRNA translation is a common feature of pathological states that are characterized by aberrant proliferation including malignancies [9]. To fulfill the high bio-energetic demands imposed by translation, the biochemical marker of cancer cells involves a shift to aerobic glycolysis, also known as the “Warburg effect” [10]. Sarcoma cells exhibit high rate of glycolysis and mRNA translation [11, 12]. Blocking energy production by glucose deprivation or glycolysis inhibitor reduces the proliferation of sarcoma cells proliferation [4, 13]. And blocking mRNA translation by cap-translation inhibitor 4EGI-1 inhibits the proliferation of sarcoma cells [14]. Thus, modulation of aberrant energy homeostasis might be a potential therapeutic strategy for sarcoma chemotherapy.

Recent studies have revealed, the mammalian target of rapamycin (mTOR) is a highly conserved Ser/Thr kinase that integrates diverse signals including nutrients, growth factors, energy and stresses to control cell growth, proliferation, survival and metabolism [15–17]. mTOR complex 1 (mTORC1), which contains mTOR, Raptor, mammalian lethal with SEC13 protein 8 (mLST8) and proline-rich Akt substrate 40 (PRAS40) [9, 18], regulates aberrant energy homeostasis through phosphorylation of eukaryotic translation initiation factor 4E-binding protein 1 (4E-BP1) [9, 19–22]. The release of phosphorylated 4E-BP1 from the mRNA m^7^-GTP cap-binding protein eIF4E promotes interaction between eIF4E and scaffolding protein eIF4G to initiate the formation of the translation-initiating complex eIF4F which is required for the cap-dependent translation of mRNAs such as c-myc. c-myc plays a crucial role in controlling glycolytic metabolism through regulating the transcription of genes involved in glycolysis [23, 24]. Therefore, mTORC1-4E-BP1 axis is thought to be central to energy homeostasis that couples cellular energy production to consumption. Since mTORC1 is highly expressed and activated in sarcoma [25, 26], the potential utility of developing energy homeostasis-targeted therapies directed toward mTORC1-4E-BP1 axis has been taken into account for the treatment of sarcoma.

*Epimedium koreanum* Nakai (Berberidaceae) traditionally used as a medicinal herb in East Asia [27]. Icariside II (IS), is an active flavonoid derived from *E. koreanum* that possesses anti-cancer effects in various cancer cells [28–30], and this indicates that, IS is a potential lead compound for anti-cancer therapy [31]. Previous studies have shown that IS inhibits both the phosphorylation of mTOR and the epidermal growth factor-induced activation of mTOR [30]. However, the effect of IS on aberrant energy homeostasis has yet to be elucidated. In this study, IS inhibited aberrant energy homeostasis evidenced by the reduction of energy production (glycolysis) and energy consumption (mRNA translation) in sarcoma cells. IS inhibited aberrant energy homeostasis through mTORC1-4E-BP1 axis, which contributed to its anti-proliferation effect. Moreover, IS suppressed mTORC1 through disrupting the assembly of mTORC1. Finally, mTORC1-4E-BP1 axis regulated the level of c-myc which linked the crosstalk between glycolysis and mRNA translation in IS treated sarcoma cells. This is a novel mechanism of IS to inhibit cell proliferation in sarcoma cells.

## RESULTS

### IS inhibits glycolysis and energy production in sarcoma cells

**Figure 1:**
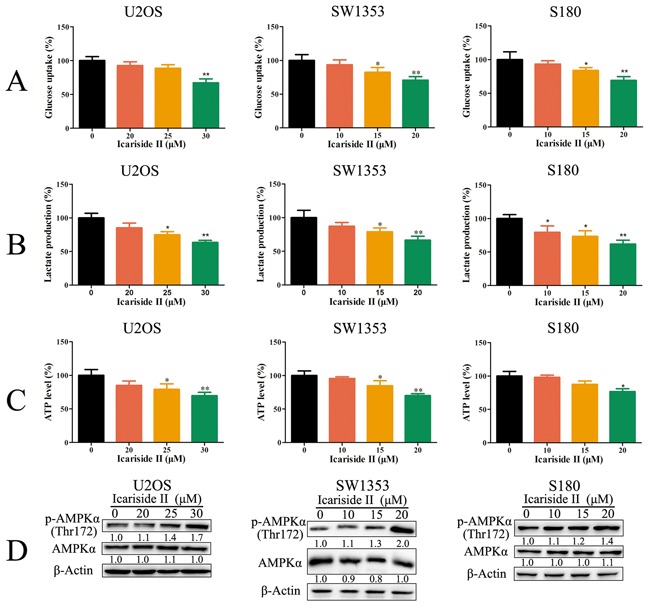
IS inhibits glycolysis and energy production in sarcoma cells **A, B.** and **C.** Sarcoma U2OS, SW1353 and S180 cells were treated with or without various concentrations of IS for 24 h. The level of glucose uptake (A), lactate production (B) and ATP production (C) were determined as described in Materials and Methods Section. **D.** The phosphorylation of AMPKα was determined by western blotting. The level of β-Actin was used as protein-loading control. Data were expressed as the mean ± S.D., n=3. *p < 0.05 and **p < 0.01 versus control group.

The level of glycolysis is always aberrantly unregulated in cancer to fulfill the high energy demands, which is needed for the rapid proliferation of cancer cells [32]. Skeletal sarcoma (such as U2OS and SW1353 cells) and soft tissue sarcoma (S180 cells) are subsets of sarcoma [33, 34]. Thus, we examined whether IS could inhibit glycolysis in sarcoma cells. High fluxes of glycolysis are distinguishing features of increased cellular uptake of glucose and abundant lactate production [35]. As shown in Figure 1A–1B, IS significantly inhibited the glycolysis rate of sarcoma cells, as manifested by the reduction of cellular lactate production and glucose consumption. ATP produced by glycolysis is required for the maintenance of cancer cellular energy homeostasis. To determine the impact of IS on the cellular energy production, ATP levels were measured. In comparison to the absent, a modest decrease in the ATP pool was detected in IS treated sarcoma cells (Figure 1C). Moreover, the energy deficit was evidenced by the increase of AMPK phosphorylation (Figure 1D). These results demonstrated that IS inhibited energy production through the suppression of glycolysis in sarcoma cells.

### IS inhibits cap-dependent translation through activation of 4E-BP1 in sarcoma cells

**Figure 2:**
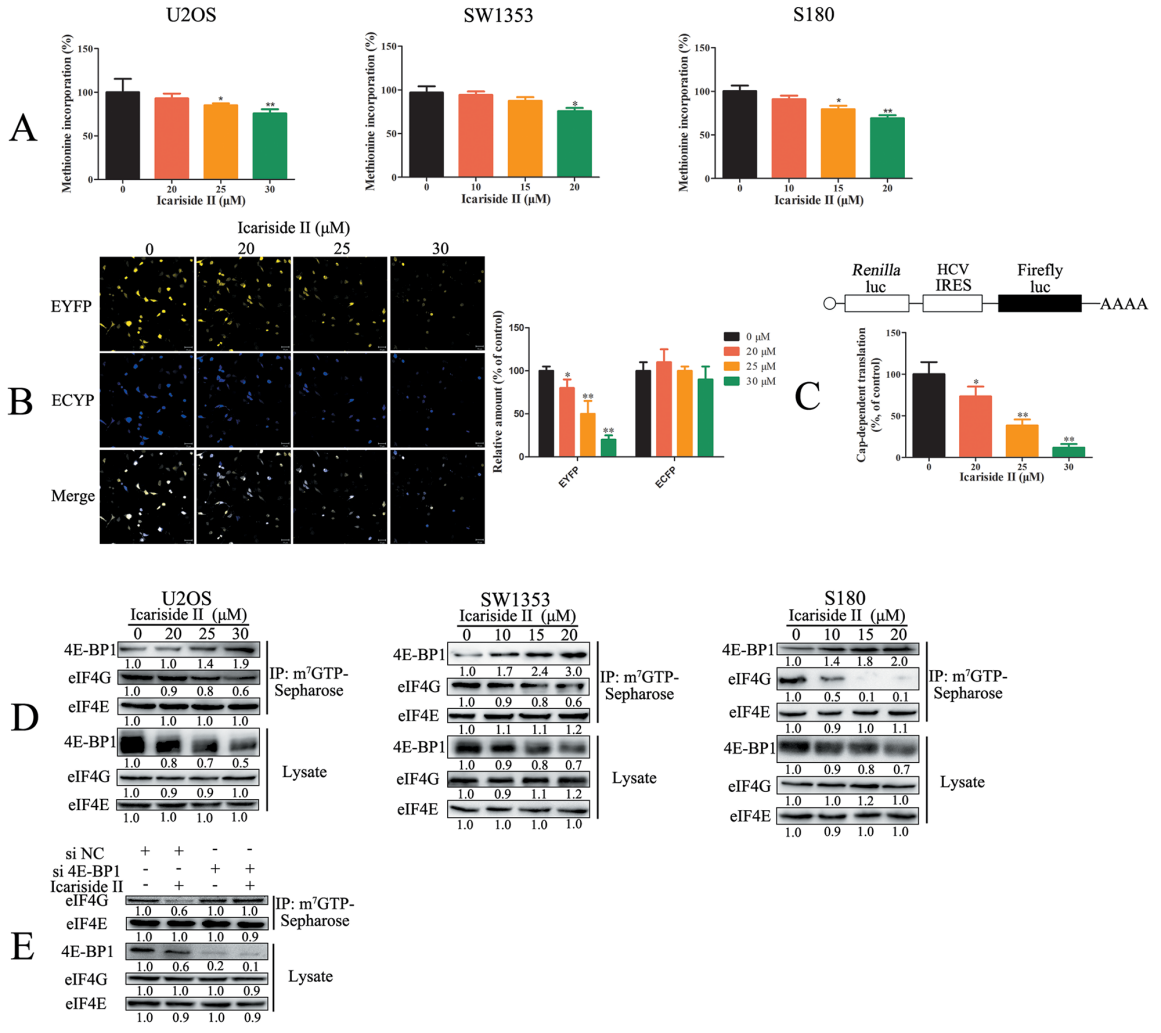
IS inhibits cap-dependent translation through activating 4E-BP1 in sarcoma cells **A.** Sarcoma U2OS, SW1353 and S180 cells were treated with IS at the indicated doses for 24 hrs, and global mRNA translation was determined by 35S-methionine assays. **B.** Sarcoma U2OS cells transfected with pYIC plasmid were treated with 0 - 30 μM IS for 24 hrs. The cellular fluorescence (EYFP and ECFP) was examined by confocal laser scanning microscope. **C.** Sarcoma U2OS cells transfected with pcDNA3-HA3 reporter plasmid were treated with 0 - 30 μM IS for 24 hrs. The luciferase activity was examined by Spectra-Max Paradigm Multi-Mode Microplate Detection Platform. **D.** Sarcoma U2OS, SW1353 and S180 cells were treated with or without various concentrations of IS for 24 hrs. m7-GTP-Sepharose affinity assay was performed to capture proteins which were tested by western blotting. **E.** Sarcoma U2OS cells interfered with 4E-BP1 or negative control (NC) siRNA were treated with or without various concentrations of IS for 24 h. m7-GTP-Sepharose affinity assay was performed to capture proteins which were tested by western blotting. Data were expressed as the mean ± S.D., n=3. *p < 0.05 and **p < 0.01 versus control group.

mRNA translation is the most energy consuming processes in cancer cells [7]. Considering the inhibition effect of IS on energy production, we evaluated the effect of IS on mRNA translation by ^35^S-methionine incorporation assay. ^35^S-methionine is incorporated into neo-synthesized proteins during mRNA translation. Thus, the detection of radioactivity is proportional to the amounts of global mRNA translation [36]. As shown in Figure 2A, IS decreased global mRNA translation in sarcoma cells, reflecting the reduction of energy consuming. Most of the translational control occurs at the rate-limiting initiation step through cap-dependent and IRES (internal ribosome entry site)-dependent pathway [37]. To determine whether IS-inhibited mRNA translation was cap-dependent or IRES-dependent, we utilized a bicistronic fluorescent reporter construct [38]. IS inhibited cap-dependent translation of yellow fluorescent protein (EYFP), but not IRES-dependent translation of cyan fluorescent protein (ECFP) (Figure 2B), indicating suggesting the selective repression of cap-dependent translation. Moreover, cap-dependent luciferase assay confirmed the effect of IS on cap-dependent translation. As shown in Figure 2C, IS significantly decreased the cap-dependent luciferase activity (Figure 2C). Cap-dependent translation involves the assembly of initiation factors (including eIF4E, eIF4A and eIF4G) to form the trimolecular cap binding complex eIF4F at the 5′ mRNA terminus, which is inhibited by the activation of 4E-BP1 [39]. To ascertain the effect of IS on capdependent translation initiation, we performed m^7^GTP-Sepharose chromatography assay which mimicked the cap structure of mRNA [40]. As a result, IS treatment caused the increase in 4E-BP1 bound to eIF4E and concurrent reduction in eIF4G binding to eIF4E, indicating that IS inhibited the assembly of eIF4F and reduced cap-dependent translation initiation in sarcoma cells (Figure 2D). Moreover, the inhibition of IS on the interaction between eIF4G and eIF4E was significantly reduced in 4E-BP1 knockdown sarcoma U2OS cells (Figure 2E), suggesting that IS inhibited cap-dependent translation initiation through 4E-BP1. These results indicated that IS inhibited cap-dependent translation through activating 4E-BP1 in sarcoma cells

### IS inhibits aberrant energy homeostasis in sarcoma cells by suppressing mTORC1 but not mTORC2

**Figure 3:**
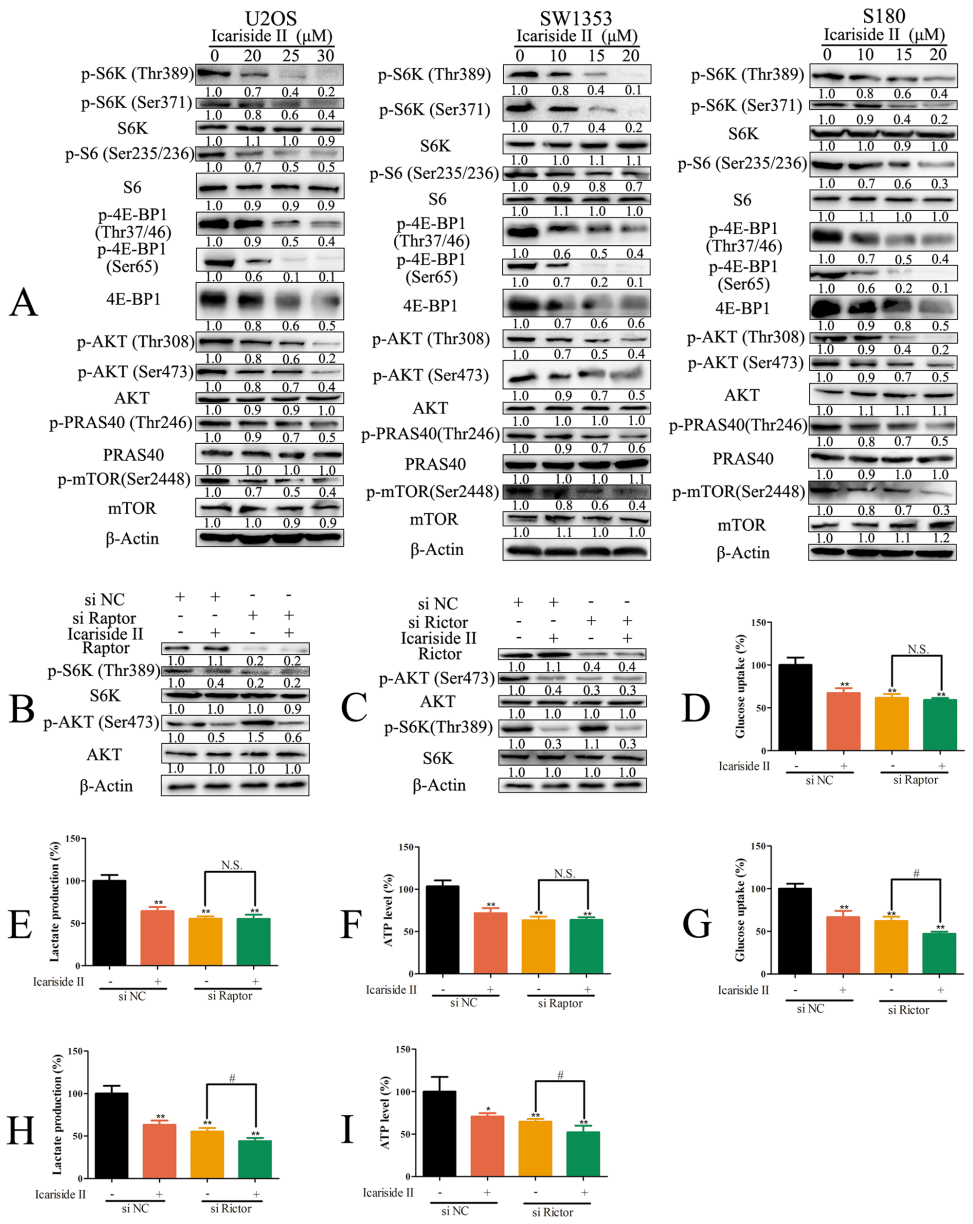
IS inhibits glycolysis in sarcoma cells by suppressing mTORC1 not mTORC2 **A.** Sarcoma U2OS, SW1353 and S180 cells were treated with IS at the indicated doses for 24 hrs. The protein extract from cells was subjected to western blotting to analyze mTORC1/2 signaling. **B.** Sarcoma U2OS cells interfered with Raptor or negative control (NC) siRNA were treated with 30 μM IS for 24 hrs. The level of raptor, p-S6K (Thr389), S6K, p-AKT (Ser473) and AKT was determined by western blotting. **C.** Sarcoma U2OS cells interfered with Rictor or NC siRNA were treated with 30 μM IS for 24 h. The level of Rictor, p-AKT (Ser473), AKT, p-S6K (Thr389) and S6K was determined by western blotting. (**D, E.** and **F.**) Sarcoma U2OS cells interfered with Raptor or NC siRNA were treated with or without 30 μM IS for 24 hrs. The level of glucose uptake (D), lactate production (E) and ATP production (F) were determined as described in Materials and Methods Section. **G, H.** and **I.** Sarcoma U2OS cells interfered with Rictor or NC siRNA were treated with or without 30 μM IS for 24 hrs. The level of glucose uptake (G), lactate production (H) and ATP production (I) were determined as described in Materials and Methods Section. Data were expressed as the mean ± S.D., n=3. *p < 0.05 and **p < 0.01 versus control group, #p < 0.05 versus absent. N.S., not significant.

mTORC1/2 signaling is central to aberrant energy homeostasis in cancer cells [9]. Thus, the effect of IS on mTORC1/2 signaling was evaluated. We found that IS repressed mTORC1/2 signaling pathways as manifest by the decreased phosphorylation levels of mTOR regulation and substrates proteins in sarcoma cells (Figure 3A). The phosphorylation of 4E-BP1 on Ser 65 residue, essential for release of 4E-BP1 from eIF4E, was significantly reduced by IS. It is reported that, through regulating its downstream targets, mTORC1 stimulates the expression of glycolytic enzymes [41]. On the other hand, Masui *et al.* identify that, the mTORC2 emerges as a particularly critical regulator of glycolysis metabolism [20]. To determine the relationship between mTORC1/2 and aberrant energy homeostasis in IS treated cells, sarcoma U2OS cells were depleted of Raptor or Rictor, which is the specific component of mTORC1 or mTORC2, respectively. Raptor depletion decreased the phosphorylation of S6K (Thr389) and increased the phosphorylation of Akt (Ser473) (Figure 3B), while Rictor knockdown reduced the phosphorylation of Akt (Ser473) and had no effect on the phosphorylation of S6K (Thr389) (Figure 3C). Raptor and Rictor knockdown both diminished the glycolysis in sarcoma U2OS cells (Figure 3D–3I). Consistent with above findings, IS had no effect on glycolysis in Raptor knockdown sarcoma U2OS cells (Figure 3D–3F), but still inhibited glycolysis in Rictor knockdown sarcoma U2OS cells (Figure 3G–3I), indicating that the suppression of mTORC1 was involved in IS inhibition of glycolysis. Taken together, these results demonstrated that IS inhibited aberrant energy homeostasis in sarcoma cells through suppressing mTORC1, but not mTORC2.

### IS inhibits mTORC1 signaling through disruption of the assembly of mTORC1

**Figure 4:**
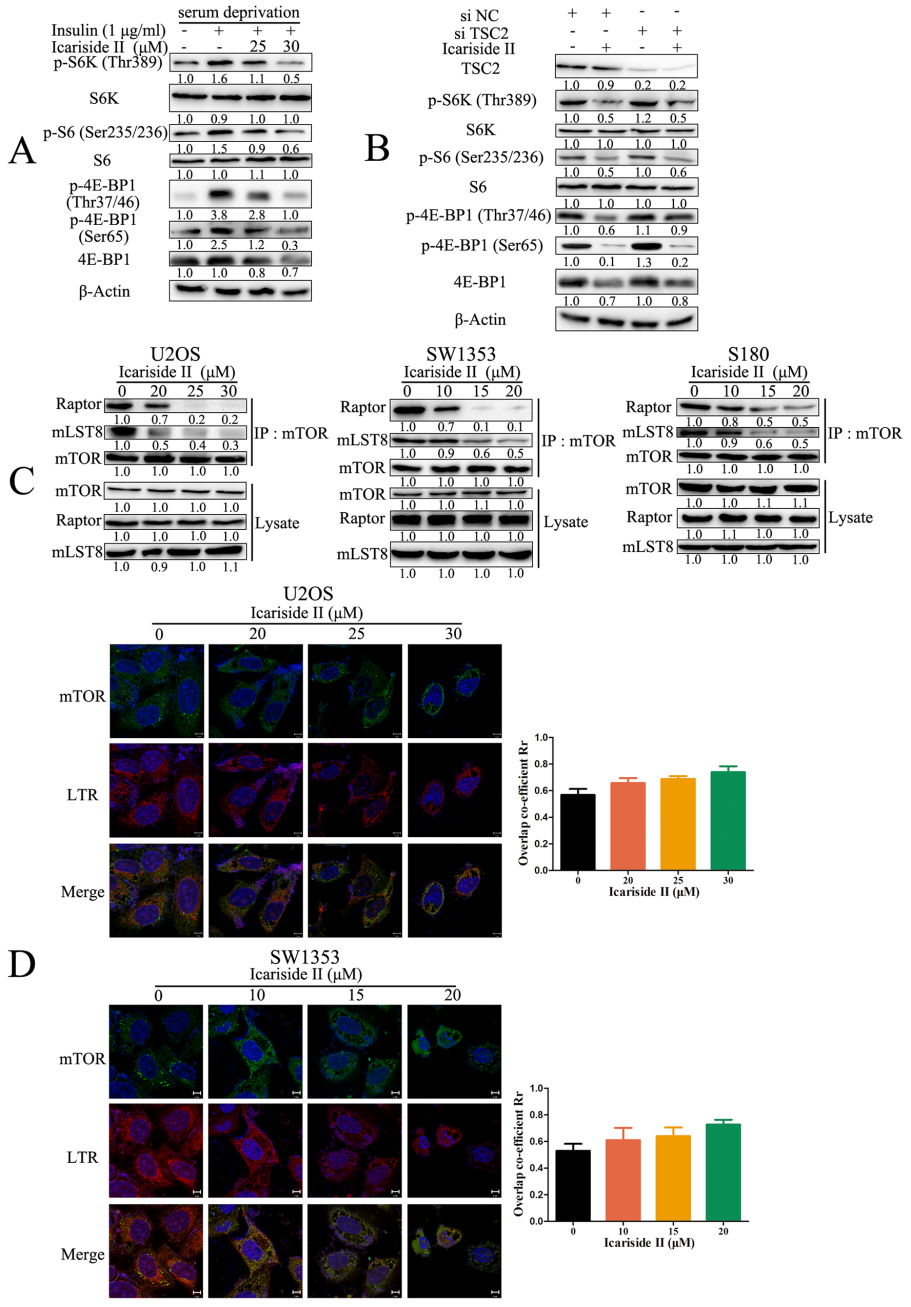
IS inhibits mTORC1 signaling through disrupting the assembly of mTORC1 **A.** Sarcoma U2OS cells were serum-starved for 18 hrs before treating with the indicated concentrations of IS for 6 hrs, followed by insulin (1 mg/mL) stimulation for another 30 min. The protein extract from the cells was subjected to western blot to analysis mTORC1 signaling. **B.** Sarcoma U2OS cells interfered with TSC2 or negative control (NC) siRNA were treated with 30 μM IS for 24 hrs. The protein extract from the cells was subjected to western blot to analyze mTORC1 signaling. The level of β-Actin was used as protein-loading control for all western blotting. **C.** Sarcoma U2OS, SW1353 and S180 cells were treated with IS at the indicated doses for 24 hrs. Cells were lysed and immunoprecipitated (IP) with mTOR antibody and the IP products were examined by western blotting. **D.** Sarcoma U2OS and SW1353 cells were treated with IS at the indicated doses for 24 hrs. The lysosomal translocation of mTORC1 was determined through evaluating the co-localization of mTOR (green) and lysosome-targetable fluorescent probe LTR (red) by confocal laser scanning microscope.

With regard to the main regulatory mechanisms of mTORC1, it has been reported that mTORC1 is activated by epidermal growth factor (EGF) and insulin through upstream signals like PI3K/Akt and Ras/RAF/ERK pathway [42, 43]. We have previously shown that IS inhibits PI3K and RAF signaling in sarcoma cells [30]. Therefore, we investigated whether these regulatory signals were also involved in the mechanism underlying IS-induced inactivation of mTORC1 signaling. As shown in Figure 4A, IS rapidly inhibited insulin-stimulated mTORC1 signaling as manifest by the reduced phosphorylation of S6K (Thr389), S6 (Ser235/236) and 4E-BP1 (Thr37/46, Ser65). Upstream signals, like PI3K and RAF, activate mTORC1 through tuberous sclerosis complex 2 (TSC2) dependent and independent pathway [44]. We found that IS still inhibited mTORC1 signaling in TSC2 knockdown sarcoma U2OS cells as indicated by the reduction of p-S6K (Thr389), p-S6 (Ser235/236) and p-4E-BP1 (Ser65) (Figure 4B), indicating that IS inhibited mTORC1 signaling mainly through TSC2-independent pathway. It is reported that dephosphorylation of PRAS40 at Thr246 disrupts the assembly of mTORC1 [45], which is necessary for mTORC1 function [46, 47]. We next examined whether IS inhibited the assembly of mTORC1 in sarcoma cells. The total levels of mTORC1 components mTOR, Raptor and mLST8 in cell lysates were not altered in IS treated sarcoma cells. However, there were less Raptor and mLST8 connected with mTOR after IS treatment, suggesting the disassembly of mTORC1 (Figure 4C). In addition to the activation of mTORC1 by upstream signal, the mTORC1 activity is also activated by the translocation of mTORC1 onto lysosomal membranes mediated by Rag small guanosine triphosphatases (GTPases) [48]. We next investigated whether lysosomal localization of mTORC1 was inhibited after IS treatment in sarcoma cells. In untreated cells, mTOR translocated to lysosomal membranes identified by the co-localization of mTOR (green) and lysosome-targetable fluorescent probe LTR (red). Similarly, IS did not disrupt the co-localization of mTOR and LTR in sarcoma cells (Figure 4D). These results indicated IS suppressed mTORC1 signaling through disruption of mTORC1 assembly in a TSC2-independent manner.

### IS inhibited glycolysis through suppressing the cap-dependent translation of c-myc in sarcoma cells

**Figure 5:**
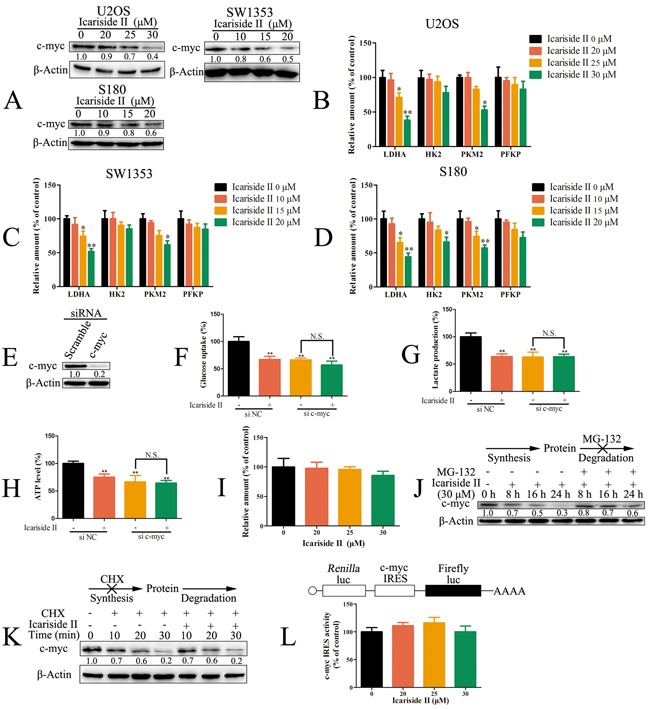
IS inhibited glycolysis through suppressing the cap-dependent translation of c-myc in sarcoma cells **A.** The level of c-myc was determined by western blotting in sarcoma U2OS, SW1353 and S180 cells with IS treatment for 24 h. **B, C.** and **D.** The level of c-myc-coded glycolytic mRNA in sarcoma U2OS (B), SW1353 (C) and S180 (D) cells exposed to IS for 24 hrs was determined by qRT-PCR. β-Actin was used as the internal control gene. **E.** Sarcoma U2OS was interfered with c-myc siRNA and the level of c-myc was determined by western blotting. (**F, G.** and **H.**) Sarcoma U2OS cells interfered with c-myc or negative control (NC) siRNA were treated with or without 30 μM IS for 24 hrs. The level of glucose uptake (F), lactate production (G) and ATP production (H) were determined as described in Materials and Methods Section. **I.** The mRNA level of c-myc was assessed by qRT-PCR in sarcoma U2OS cells exposed to IS for 24 hrs. β-Actin was used as the internal control gene. **J.** Western blotting analysis of c-myc in sarcoma U2OS cells treated with IS (30 μM) after 1 hr MG132 pretreatment. The level of β-Actin was used as protein-loading control for all western blotting. **K.** Western blotting analysis of c-myc in sarcoma U2OS cells treated with CHX after 8 hrs IS (30 μM) treatment. The level of β-Actin was used as protein-loading control for all western blotting. **L.** Sarcoma U2OS cells transfected with pRMF reporter plasmid were treated with 0 - 30 μM IS for 24 hrs. The luciferase activity was examined by Spectra-Max Paradigm Multi-Mode Microplate Detection Platform. Data were expressed as the mean ± S.D., n=3. *p < 0.05 and **p < 0.01 versus control group, #p < 0.05 versus absent.

It has been demonstrated that the cellular level of c-myc controls glycolysis metabolism [49], through regulating the transcription of genes involved in glycolysis [23, 24]. In our study, the level of c-myc was decreased after IS treatment in sarcoma cells (Figure 5A). We measured the mRNA levels of c-myc-related glycolytic pathway components. As shown in Figure 5B–5D, IS significantly decreased the mRNA level of c-myc-coded glycolytic enzymes like *LDHA* and *PKM2 in U2OS* and SW1353 cells as well as *LDHA*, *HK2* and *PKM2* in S180 cells. To address whether the suppression of c-myc mediated IS inhibition of glycolysis in sarcoma cells, c-myc expression was silenced by siRNA (Figure 5E). As expected, the depletion of c-myc markedly reduced both the glucose uptake and lactate production. Furthermore, the glycolysis was not significantly regulated by IS treatment in c-myc knockdown sarcoma U2OS cells (Figure 5F–5H). To ascertain whether the reduction of c-myc induced by IS occurred at transcriptional level, we analyzed the level of c-myc mRNA in IS treated sarcoma U2OS cells. Real-time PCR indicated that the level of c-myc mRNA was not significantly changed after IS treatment (Figure 5I). We next investigated the effect of IS on the translation of c-myc mRNA. Sarcoma U2OS cells were pre-treated with proteasome inhibitor MG132 to exclude the influence of protein degradation. Although c-myc expression was substantial increased in MG132 treated cells, IS still effectively decreased the level of c-myc protein (Figure 5J), confirming that reduction in the c-myc mRNA translation was correlated with IS inhibition of c-myc protein. To assess the influence of IS on the degradation of c-myc protein, sarcoma U2OS cells were treated with the translation inhibitor cycloheximide (CHX) after 8 h exposure to IS. As shown in Figure 5K, the degradation rates of c-myc were similar in both IS-treated and untreated cells, suggesting that the increase of its protein degradation was not involved in IS inhibition of c-myc. These results indicated that the suppression of c-myc mRNA translation contributed to the decreased synthesis rate of its protein induced by IS. The cap-dependent translation was reduced by IS treatment in sarcoma cells (Figure 2). To evaluate whether the inhibition of IRES-dependent translation was involved in the inhibition of c-myc mRNA translation, sarcoma U2OS cells were transfected with the dicistronic plasmid, which contains the *c-myc* IRES between *Renilla* and firefly luciferase genes. The data showed that *c-myc* IRES activity was not inhibited by IS, indicating that IS suppressed c-myc mRNA translation in a cap-dependent manner (Figure 5L). Taken together, our data demonstrated that, the IS inhibited glycolysis through suppressing the cap-dependent translation of c-myc in sarcoma cells.

### IS inhibits c-myc-regulated glycolysis through mediating mTORC1-4E-BP1 axis in sarcoma cells

**Figure 6:**
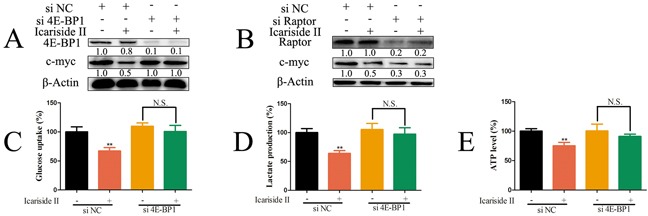
IS inhibits c-myc-regulated glycolysis through mediating mTORC1-4E-BP1 axis in sarcoma cells **A.** Sarcoma U2OS cells interfered with 4E-BP1 or negative control (NC) siRNA were treated with 30 μM IS for 24 hrs. The level of 4E-BP1 and c-myc protein was tested by western blotting. **B.** Sarcoma U2OS cells interfered with Raptor or NC siRNA were treated with 30 μM IS for 24 hrs. The level of Raptor and c-myc was determined by western blotting. The level of β-Actin was used as protein-loading control for all western blotting. (**C, D.** and **E.**) Sarcoma U2OS cells interfered with 4E-BP1 or NC siRNA were treated with or without 30 μM IS for 24 hrs. The level of glucose uptake (C), lactate production (D) and ATP production (E) were determined as described in Materials and Methods Section. Data were expressed as the mean ± S.D., n=3. *p < 0.05 and **p < 0.01 versus control group. N.S., not significant.

It has been proved that hyperactivated eIF4F results in stimulating translation of a subset of mRNAs including c-myc mRNA [50], characterized by lengthy, G-C rich and highly structured 5-UTRs [51]. It is well documented that mTORC1-4E-BP1 axis is critical for eIF4F activation, which is also associated with increased cellular proliferation [21, 52]. We tested whether mTORC1-4E-BP1 axis was involved in IS inhibition of c-myc. As shown in Figure 6A–6B, IS had no effect on the level of c-myc in 4E-BP1 or Raptor knockdown sarcoma U2OS cells. Moreover, 4E-BP1 knockdown abolished the effect of IS on the inhibition of glycolysis and ATP production (Figure 6C–6E) in sarcoma U2OS cells. Overall, these observations indicated a distinct cross-talk between mTORC1-4E-BP1 axis and c-myc-regulated glycolysis in sarcoma cells.

### Inhibiting aberrant energy homeostasis contributes to the anti-proliferation effect of IS in sarcoma cells

**Figure 7:**
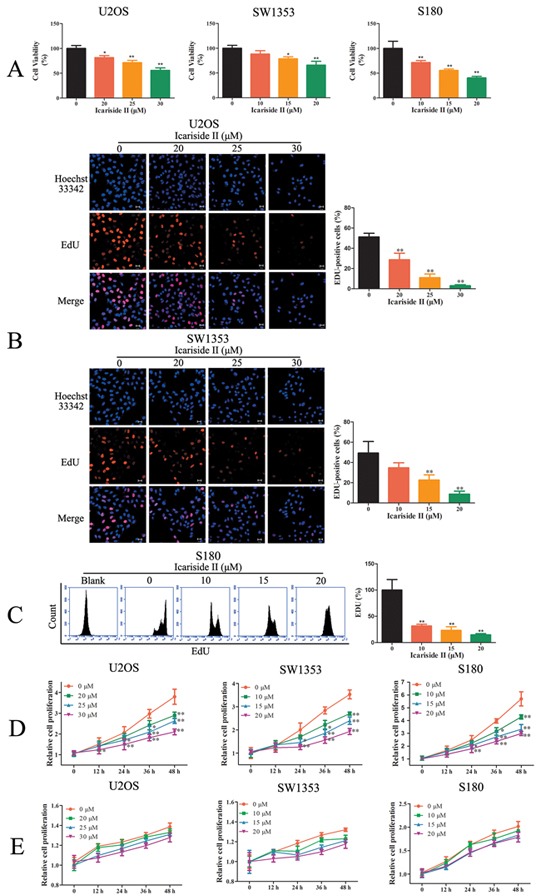
Inhibiting aberrant energy homeostasis contributed to the anti-proliferation effect of IS in sarcoma cells **A.** Sarcoma U2OS, SW1353 and S180 cells were treated with IS for 24 hrs. The cell viability was determined by MTT assay. **B.** U2OS and SW1353 cells were incubated with variable concentrations of IS for 24 hrs. EdU staining was then performed and the cells were observed by confocal laser scanning microscope. **C.** S180 cells were incubated with variable concentrations of IS for 24 hrs. EdU staining was then performed and the cells were determined by flow cytometer. **D.** The growth curves of IS treated sarcoma U2OS, SW1353 and S180 cells cultured in media containing glucose were determined by a Countess Automated Cell Counter to count the number of Trypan Blue-negative cells. **E.** The growth curves of IS treated sarcoma U2OS, SW1353 and S180 cells cultured in media containing galactose were determined by a Countess Automated Cell Counter to count the number of Trypan Blue-negative cells. Data were expressed as the mean ± S.D., n=3. *p < 0.05 and **p < 0.01 versus control group.

Aberrant energy homeostasis is a common mediator in cancer which is positively correlated with proliferation rates [9, 53]. Since the aberrant energy homeostasis was injured by IS treatment, we next investigated the effect of IS on the proliferation of sarcoma cells. IS inhibited cell viability in sarcoma cells (Figure 7A), but exhibited little cytotoxicity in normal osteoblast hFOB 1.19 cells (Supplementary Figure 1). EDU incorporation assay also confirmed that IS inhibited the proliferation of sarcoma cells (Figure 7B–7C). We then evaluated the role of inhibited energy homeostasis in IS inhibition of cell proliferation. IS suppressed the proliferation of sarcoma cells growing in the medium containing glucose. The effect of which became apparent by 24 h, and with increasing magnitude of effect by 48 h (Figure 7D). Next, cells were incubated in the media containing galactose instead of glucose, thereby reducing glycolytic flux and forcing the cell to rely on mitochondrial oxidative phosphorylation. Under these conditions, the aberrant energy homeostasis was reduced evidenced by the lower proliferation rates (Figure 7D–7E). The inhibition of IS on proliferation rate was partly diminished (Figure 7E), demonstrating that IS regulated the proliferation of sarcoma cells partially through inhibiting aberrant energy homeostasis.

### IS inhibits the growth of sarcoma cells *in vivo* through suppressing mTORC1-4E-BP1 axis mediated energy homeostasis

**Figure 8:**
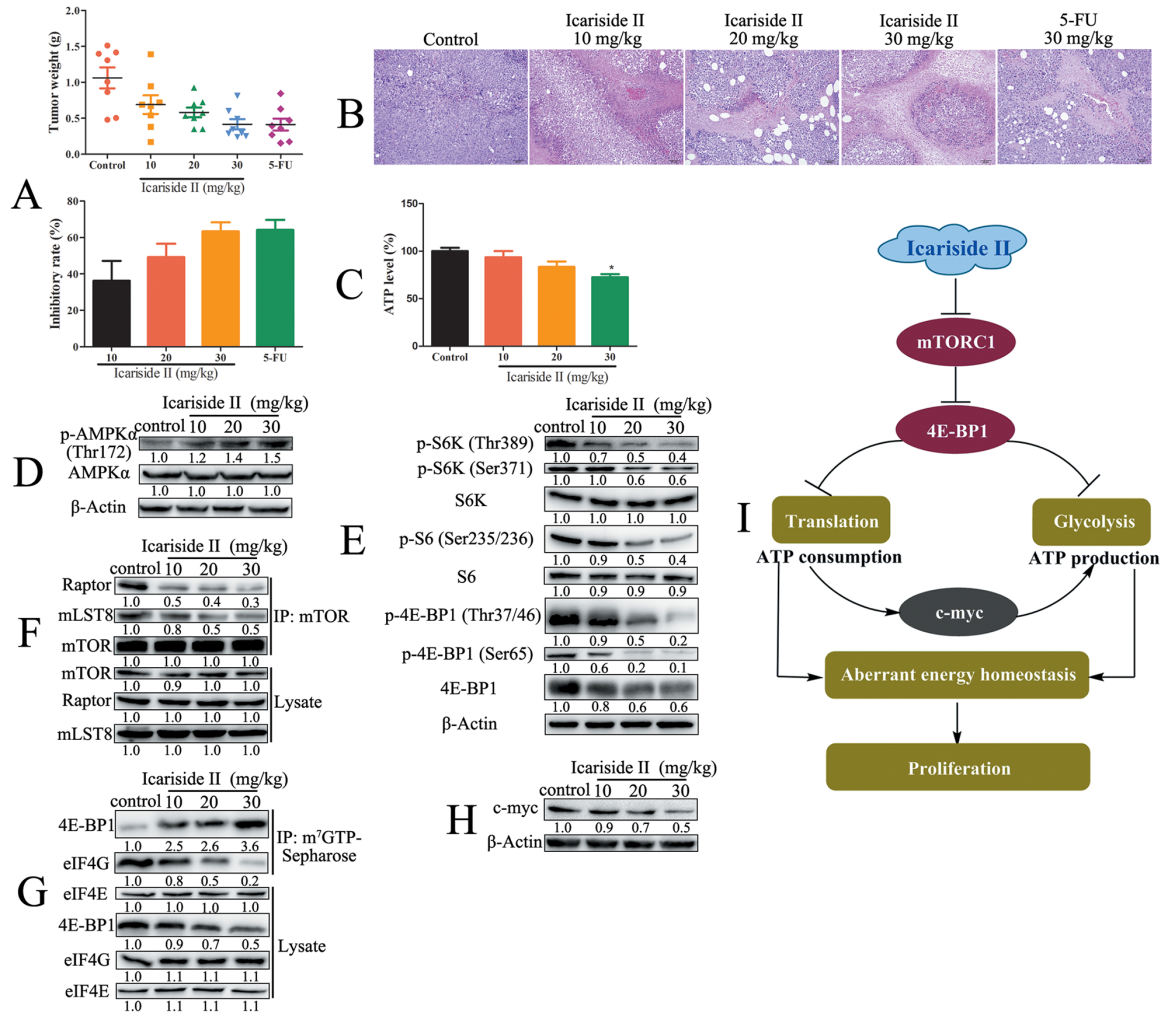
IS inhibits the growth of sarcoma cells *in vivo* through suppressing mTORC1-4E-BP1 axis mediated energy homeostasis **A.** The mice bearing sarcoma S180 cell-derived tumors were treated with or without various concentrations of IS or 5-FU for 8 d. The weight of tumors was recorded and the inhibition rate was estimated (n=8). **B.** The tumors from treated and control mice were stained with H&E to evaluate the anti-tumor effect of IS. **C.** The ATP production was determined as described in Materials and Methods Section. **D.** The phosphorylation of AMPKα was determined by western blotting. **E.** The downstream proteins of mTORC1 signaling were determined by western blotting. The level of β-Actin was used as protein-loading control for all western blotting. **F.** The assembly of mTORC1 was determined by co-immunoprecipitation. **G.** The cap-dependent translation was determined by m^7^-GTP-Sepharose affinity assay and the capture proteins were tested by western blotting. **H.** The level of c-myc was determined by western blotting. Data were expressed as the mean ± S.D., n=3. *p < 0.05 and **p < 0.01 versus control group. **I.** Schematic representation of IS inhibition of aberrant energy homeostasis in sarcoma cells.

To evaluate the anti-tumor effect of IS on sarcoma cells *in vivo*, mouse sarcoma S180 cell-derived tumor model was used. *In vivo* experiments showed that, the IS treatment inhibited the tumor weight (Figure 8A). After 8 days treatment, IS (30, 20 and 10 mg/kg) showed significantly inhibitory effect on the growth of inoculated S180 cells in mice. The weight of excisional tumors from IS treated group was significantly lower as measured by 0.43 ± 0.19 g, 0.56 ± 0.20 g and 0.77 ± 0.31 g, respectively, compared to control group as 1.15 ± 0.41 g. H&E staining confirmed the inhibitory effect of IS on S180 tumor progression, as mentioned that IS caused disruption of normal compact architecture of tumor tissue as compared with the untreated control (Figure 8B). Moreover, the mice treated with IS had a stable body weight without morphological changes in heart, liver, spleen, lung and kidney (Supplementary Figure S2). We further demonstrated that IS inhibited energy production as evidenced by the decreased ATP level and the increase of AMPK phosphorylation *in vivo* (Figure 8C–8D). The inhibition of mTORC1 signaling by IS was also evidenced by decreased phosphorylation levels of S6K (Thr389), S6 (Ser235/236), 4E-BP1 (Thr37/46, Ser65) (Figure 8E), as well as the disassembly of mTORC1 (Figure 8F). The data from m^7^GTP-Sepharose chromatography assay showed that IS disrupted cap-dependent translation *in vivo* (Figure 8G). In addition, IS decreased the protein level of c-myc in S-180 tumors (Figure 8H). These results further confirmed that IS inhibited the growth of sarcoma cells through suppressing mTORC1-4E-BP1 axis mediated energy homeostasis (Figure 8I).

## DISCUSSION

mTORC1 is recognized as a key metabolic sensor that integrates nutrient (amino acids), the cellular energy status (high ATP/AMP ratio) and growth factor levels (for example, insulin and EGF), by promoting protein synthesis and activating bioenergetic processes [54]. IS is found to be a natural mTOR inhibitor [30]. However, the effects and underling mechanisms of IS on mTORC1 and its mediated aberrant energy homeostasis have not been elucidated. Herein, we reported that, the IS inhibited mTORC1 signaling in sarcoma cells (Figure 3A). The oncogenic PI3K/AKT and Ras/ERK pathway, which stimulated by various cell-surface receptors (like EGFR, IGFR), activate mTORC1 by TSC2-dependent and -independent pathway [45, 55, 56]. Studies have shown that, IS suppresses the PI3K/Akt and Ras/ERK pathways *in vitro* and *in vivo*. Moreover, IS alleviates EGF-stimulated phosphorylation of mTOR [30]. Consistently, in this report, IS blocked insulin-activated mTORC1 signaling (Figure 4A). Further study indicated that IS inhibited mTORC1 signaling in a TSC2-independent manner (Figure 4B). IS was shown to inhibit the phosphorylation of not only Akt but also PRAS40 at the Akt target site Thr246 (Figure 3A). It has been reported that, the hypo-phosphorylated PRAS40 binds not only to raptor via the TOS motif, but also to mTOR-Raptor via the KSLP region, in order to disrupt the assembly of mTORC1. Dephosphorylation of PRAS40 at Thr246 promotes PRAS40 inhibition of mTORC1 [57]. Thus, the dephosphorylation of PRAS40 by IS in sarcoma cells *in vitro* (Figure 3A) and *in vivo* [30], contributed to the disassociation of mTOR-Raptor complex (Figure 4C). Unlike growth factors, amino acids stimulate mTORC1 activation through promoting its lysosome translocation [58]. Our previous study indicates that IS induces lysosome malfunction through altering lysosomal membrane and suppressing lysosomal acidification [59], indicating that IS may affect the lysosome translocation of mTORC1. However, IS did not block the lysosome localization of mTORC1 (Figure 4D). Above all, IS suppressed mTORC1 signaling through PRAS40 induced disruption of mTORC1 assembly.

Increased cell proliferation correlates with the elevated mRNA translation in cancer. mRNA translation includes initiation, elongation and termination. Although all steps of mRNA translation are highly regulated in cancer cells, most of the translational control occurs at the rate-limiting initiation step [60]. In cancer cells, the majority of mRNAs are translated in a cap-dependent manner, relying on the assembly of eIF4F complex at the 7-methyl-guanosine cap at the 5′ end of an mRNA transcript [51]. In this study, IS suppressed eIF4F related cap-dependent translation initiation, leading to the reduction of global mRNA translation (Figure 2A–2D, 8G). 4E-BP1, the important substrate of mTORC1, regulates cap-dependent translation initiation in a phosphorylation-dependent manner [61]. Hypo-phosphorylated 4E-BP1 binds to eIF4E and prevents its assembly into the eIF4F complex, thus blocking cap-dependent mRNA translation initiation. In line with the above findings, the phosphorylation of 4E-BP1 (Thr 37/46, Ser65) was decreased after IS treatment (Figure 3A). Moreover, our results demonstrated that 4E-BP1 acted as a major mediator of the effects of mTORC1 on cap-dependent translation initiation. Knockdown of 4E-BP1 completely reversed the suppression of IS in cap-dependent translation initiation (Figure 2E). Based on these results, we suggested that, IS suppressed cap-dependent translation through inhibiting mTORC1-4E-BP1 axis.

To maintain enhanced proliferation and growth rates, cancer cells shift their bio-energetic state to glycolysis to boost energy production. ATP generated from glycolysis is undoubtedly important for energy demand and plays an important role in biosynthesis for cancer cells. Growing evidence proves that, the mTORC1-4E-BP1 axis is well positioned to act as a central node of glycolysis [41, 62]. In TSC2-deficient cells, the hyperactivation of mTORC1 increases the transcription of glycolytic enzymes and promotes glycolysis, which is blocked by the mTORC1 inhibitor rapamycin [41]. In this study, IS decreased glycolysis, leading to the energy deficit in sarcoma cells (Figure 1, 8C–D). Moreover, our results indicated that, the suppression of mTORC1 was also involved in IS inhibition of glycolysis (Figure 3D–3F). mTORC1 controls bio-energetics state through hyper-phosphorylation of 4E-BP1 [21]. Lacking 4E-BP1 induces resistance to mTORC1 inhibitor [63]. Consistently, our results proved that the 4E-BP1 knockdown U2OS cells were insensitive to IS treatment (Figure 6C–6E), suggesting that mTORC1-4E-BP1 axis was correlated with IS inhibition of glycolysis.

mTORC1-4E-BP1 axis acted as a central node of cellular networks that coordinated mRNA translation and glycolysis in IS-treated sarcoma cells (Figure 1–4). It is necessary to clarify that, the link between the perturbations in mRNA translation and glycolysis. c-myc, an transcription factor that promotes the transcription of glucose transporters and glycolytic enzymes [24]. c-myc increases the transport of glucose, its catabolism to pyruvate, and ultimately to lactate. In this study, IS decreased the protein expression of c-myc (Figure 5A), accompanied with the down-regulation of c-myc-coded mRNA level of glycolytic enzymes (Figure 5B–5D). Furthermore, IS failed to inhibit glycolysis in c-myc knockdown sarcoma U2OS cells (Figure 5F–5H), suggesting the possibility that IS inhibited glycolysis through suppressing c-myc. c-myc mRNA contains long, highly structured 5′ UTR [64] and expected to be highly dependent on eIF4F for efficient translation [50], which is mainly regulated by mTORC1-4E-BP1 axis. The transcription and degradation of c-myc were not significantly influenced by IS treatment. Next, IS treatment did not notably alter the IRES activity of c-myc (Figure 5J–5L). Furthermore, the level of c-myc was insensitive to IS in 4E-BP1 or Raptor knockdown cells (Figure 6A–6B), indicating that, IS inhibited the protein expression of c-myc by suppressing mTORC1-4E-BP1 axis mediated cap-dependent translation. Taken together, our data demonstrated that, c-myc acted as a critical link of the effects of mTORC1-4E-BP1 axis on mRNA translation and glycolysis.

IS-induced impairment in energy production (glycolysis) was accompanied by the reduction in energy consumption (mRNA translation), which results in a state of metabolic quiescence. These data suggested that IS inhibited aberrant energy homeostasis and induced a state of “metabolic dormancy” in sarcoma cells, which contributed to its anti-proliferation effect (Figure 7–8). IS suppressed mTORC1 through disrupting the assembly of mTORC1. We found that IS inhibited aberrant energy homeostasis through regulating mTORC1-4E-BP1 axis in sarcoma cells. Moreover, c-myc mediated the crosstalk between mRNA translation and glycolysis in IS treated sarcoma cells (Figure 8I). Taken together, these findings suggested a potential clinical application of IS in sarcoma therapy and provided a new mechanism involving the inhibition of aberrant energy homeostasis via mTORC1-4E-BP1 axis to enlighten the inhibition effect of IS on sarcoma cells.

## MATERIALS AND METHODS

### Materials

IS (purity > 99%) was dissolved by dimethyl sulfoxide (DMSO). The DMSO concentration in all drug-treated cells was less than 0.1 %. MTT, 5-FU and Insulin were purchased from Sigma (St Louis, MO). MG-132 was purchased from Beyotime (Haimen, China). CHX was purchased from Biovision (San Francisco, USA). All antibodies were purchased from Cell Signaling Technology (Danvers, MA).

### Cell culture

Sarcoma U2OS cells (Human osteosarcoma), SW1353 cells (Human chondrosarcoma) and S180 cells (mouse fibrosarcoma) were purchased from Cell Bank of Shanghai Institute of Biochemistry and Cell Biology, Chinese Academy of Sciences (Shanghai, China). U2OS and S180 cells were cultured in RPMI 1640 media containing 10 % heat-inactivated fetal bovine serum (FBS) and incubated at 37°C with 5 % CO_2_. SW1353 cells were cultured in DMEM media containing 10 % FBS and incubated at 37°C with 5 % CO_2_.

### Glucose uptake assay

U2OS or S180 cells were planted in 6-well plates (2 × 10^5^ cells/well). After incubation over night, RPMI 1640 medium (11 mM glucose) containing 3 % FBS with or without serial concentrations of IS were replaced to the 6-well plates. After cells were treated with IS for 24 h, culture media were collected and diluted 1 : 1000 in water. Glucose in the culture media was quantitated via an Amplex Red Glucose/Glucose Oxidase Kit (Life Technologies, Carlsbad, USA) using a standard curve prepared with serial dilutions of RPMI 1640 (11 mM glucose) into glucose-free RPMI 1640. Fluorescence was read using a Spectra-Max Paradigm Multi-Mode Microplate Detection Platform (Molecular Devices, Sunnyvale, California, United States) at Ex./Em. = 530 nm / 590 nm and normalized to the number of cells in each well counted by BD Accuri™ C6 flow cytometer (Becton & Dickinson Company, Franklin Lakes, NJ). Cell-free medium was used as a background control. The concentration of glucose uptake in each sample was then calculated. Glucose uptake was determined by subtracting the amount of glucose in each sample from the total amount of glucose in cell-free medium (11 mM glucose). In SW1353 cells, the incubation medium was replaced by DMEM medium and glucose uptake was assayed by the same method.

### Lactate generation assay

U2OS or S180 cells were cultured in 6-well plates (2 × 10^5^ cells per well). After incubation over night, RPMI 1640 medium containing 3 % FBS with or without serial concentrations of IS were replaced to the 6-well plates and incubated for 24 hrs after which, culture media were collected. Lactate generation was assayed using the Lactic Acid production Detection kit (KeyGen, Nanjing, China) following the manufacturer's instructions. Absorbance was determined by Molecular Devices SpectraMax Plus 384 microplate reader (Molecular Devices, Sunnyvale, CA, USA) at 570 nm, and normalized to the number of cells in each well counted by BD Accuri™ C6 flow cytometer (Becton & Dickinson Company, Franklin Lakes, NJ). Amount of lactate generation was calculated as follows: lactate generation (mM) = 3 × (OD_sample normalized_ – OD_control_)/(OD_standard_ − OD_control_). In SW1353 cells, the incubation medium was replaced by DMEM medium and lactate generation was assayed by the same method.

### ATP assay

U2OS or S180 cells were cultured in 6-well plates (2 × 10^5^ cells per well). After incubation over night, RPMI 1640 medium containing 3% FBS with or without serial concentrations of IS were replaced to the 6-well plates and incubated for 24 hrs after which, the level of ATP in the cells was measured using the ATP Bioluminescence Assay Kit (Beyotime, Haimen, China) following the manufacturer's instructions. Briefly, cells were harvested and lysed with a lysis buffer, followed by centrifugation at 10,000 × g for 5 min at 4°C. The level of ATP was determined by mixing 50 μL of the supernatant with 50 μL of luciferase reagent, which catalyzed the light production from ATP and luciferin. The emitted light was linearly related to the ATP concentration and measured using a Spectra-Max Paradigm Multi-Mode Microplate Detection Platform (Molecular Devices, Sunnyvale, California, United States) which was normalized to the protein concentration in each well. In SW1353 cells, the incubation medium was replaced by DMEM medium and ATP was assayed by the same method.

### RNA interference

Small interfering RNA (siRNA) was synthesized by Biomics Co. (Nantong, China) which consisted of a pool of three siRNA each interfered the mRNA encoding Raptor, Rictor, TSC2, c-myc or 4E-BP1 for maximum knockout efficiency. Briefly, U2OS cells were grown in 6-well plates and transfected using Lipofectamine 2000 (Life technologies, Carlsbad, USA) with siRNA for Raptor, Rictor, TSC2, c-myc or 4E-BP1 or the scrambled control at a final concentration of 100 nM according to the manufacturer's instructions. Twenty-four hours later, cells were treated with or without IS (25 - 30 μM) for 24 h.

### Western blotting

For western blot analysis, U2OS, SW1353 and S180 cells in 6-cm dish were treated with or without IS for 24 h. The cells were lysed as previously described [65]. Equal amounts of proteins were separated on 8 % - 15 % SDS-PAGE depending on the molecular sizes of the proteins and blotted onto a nitrocellulose membrane (BioRad Laboratories, Hercules, CA) as previously described [65]. The band intensity was measured with Image Lab 4.0 ((BioRad Laboratories, Hercules, CA).

### Co-immunoprecipitation

U2OS, SW1353 and S180 cells in 6-cm dish were treated with or without IS for 24 h. For co-immunoprecipitation, cells were lysed in ice-cold CHAPS containing lysis buffer and proteins were precipitated from the supernatant by the addition of mTOR antibody (cell signaling technology, 1:100) and 50 μL 50% protein A+G Sepharose according to previous study with some modifications. Proteins were denatured by the addition of 50 μL of sample loading buffer and boiled for 10 min, resolved by SDS-PAGE, and finally analyzed by *Western blotting*.

### Immunofluorescence

U2OS and SW1353 cells in 6-cm dish were treated with or without IS for 24 hrs. The cells were fixed with 4% paraformaldehyde in PBS at 15 min intervals, permeabilized with ice-cold methanol at -20°C and blocked with 5 % BSA for 1 h. Incubation with primary antibodies against mTOR (Cell Signaling Technology, Danvers, MA) was done overnight at 4°C. The cells were then incubated with Alexa Flour 488-conjugated secondary antibody for 2 hrs at room temperature in the dark. The lysosomes were stained with Lyso-Tracker Red (LTR) (Life technologies, Carlsbad, USA) for 45 min and the nuclei were stained with Hoechst 33342 (Beyotime, Haimen, China) 30 min before imaging. A laser scanning confocal microscope LSM 700 (Carl Zeiss, Oberkochen, Germany) was used for co-localization analysis.

### ^35^S-methionine incorporation assay

The global mRNA translation were assessed by a Promix ^35^S-methionine pulse assay, as reported [36]. U2OS and S180 cells were incubated for 2 h, at 10^5^/mL in a low methionine medium (80 % RPMI 1640 media without methionine, 10 % MEM, with 10 % dialysed FBS), without or with IS, then labeled for 45 min with ^35^S-methionine (33 μ Ci). The amount of radioactivity incorporated into proteins was determined by trichloroacetic acid precipitation. In SW1353 cells, the incubation medium was replaced by DMEM medium and ^35^S-methionine incorporation was assayed by the same method.

### Cap-dependent reporter gene assay

pYIC is a gift from Han Htun (Addgene plasmid # 18673) which produces a bi-cistronic mRNA encoding EYFP and ECFP. EYFP translation depends on 5′cap sequence and ECFP translation depends on IRES [38]. If a protein interferes with the cap-dependent pathway, EYFP translation is reduced without the reduction of ECFP translation. Transfection of pYIC plasmids was performed with U2OS cells grown in 35-mm glass-bottom dishes (Shengyou Biotechnology, Hangzhou, China). Cells were transfected with 0.5μg DNA and 0.3 μL Lipofectamine 2000 (Life technologies, Carlsbad, USA). After 2 hrs, the medium was replaced with fresh medium. Twenty-four hours later, the medium was replaced with fresh medium containing IS at different concentrations (20 - 30 μM) or DMSO as vehicle (< 0.1 %). After 24 hrs of administration, cellular fluorescence (YFP/CFP) was examined on a confocal laser scanning microscope LSM 700 (Carl Zeiss, Oberkochen, Germany).

### Luciferase activity assay

pcDNA3-HA3 reporter plasmid was constructed based on Suh's plasmid with modification [66]. U2OS cells were seeded in 24-well plates and transfected with the pcDNA3-HA3 reporter plasmid using Lipofectamine 2000 (Life technologies, Carlsbad, USA). Twenty-four hours later, the medium was replaced with fresh medium containing IS at different concentrations (20 - 30 μM) or DMSO as vehicle (< 0.1 %). After 24 hrs of administration, the cells were lysed and subjected to a luciferase activity assay (Beyotime, Haimen, China) using Spectra-Max Paradigm Multi-Mode Microplate Detection Platform (Molecular Devices, Sunnyvale, California, United States). Luciferase activity is normalized to the luciferase value obtained for pcDNA3-HA3 in the absence of treatment.

### c-myc IRES activity assay

pRMF is a gift from Y Shi [67]. U2OS cells were seeded in 24-well plates and transfected with the pRMF reporter plasmid using Lipofectamine 2000 (Life technologies, Carlsbad, USA). Twenty-four hours later, the medium was replaced with fresh medium containing IS at different concentrations (20 - 30 μM) or DMSO as vehicle (< 0.1 %). After 24 hrs of administration, the cells were lysed. The activity of firefly and Renilla luciferases in lysates were measured using a dual-luciferase reporter assay system (Promega) using Spectra-Max Paradigm Multi-Mode Microplate Detection Platform (Molecular Devices, Sunnyvale, California, United States). Luciferase activity is normalized to the luciferase value obtained for pRMF in the absence of treatment.

### Cap (m^7^-GTP) pull down assay

U2OS, SW1353 and S180 cells in 6-cm dish were treated with or without IS for 24 h. Total of 700 μg of cellular proteins in lysis buffer (20 mM Tris-HCl, pH 7.5, 150 mM NaCl, 1 mM ethyleneglycolbis (aminoethylether)-tetraacetic acid, 1 % Triton, 1 mM ethylenediaminetetraacetic acid, 2.5 mM sodium pyrophosphate, 1 mM b-glycerophosphate, 1 mM Na_3_VO_4_ and 1 μg/ml leupeptin) was mixed with 50 μl of 7-methyl-GTP-Sepharose-4B bead suspension (GE Healthcare) and incubated overnight. After washing the pellet, the affinity complex was denatured by the addition of 50 μL of sample loading buffer and boiled for 10 min, resolved by SDS-PAGE, and finally analyzed by *Western blotting*.

### Quantitative real-time PCR (qRT-PCR) analysis

Total RNA samples from U2OS, SW1353 and S180 cells were extracted using RNAiso Plus reagent following the manufacturer's protocols. RNA (1 μg) was reverse-transcribed using a ReverTra Ace qPCR RT-Kit (Toyobo Life Science, Osaka, Japan) in a MyCycler PCR system (BioRad Laboratories, Hercules, CA). SYBR Green PCR Master Mix was purchased from Toyobo Life Science. The 2^−ΔΔCT^ cycle threshold method was used for the calculation of relative differences in mRNA abundance with a LightCycler 480 qPCR System (Roche Molecular Biochemicals, Mannheim, Germany). Data were normalized to the expression of *β*-actin. The results of real-time PCR were expressed as fold-changes. The normalized value of the target mRNA of the control group is arbitrarily presented as 1. The pairs of primer for PCR were listed below:

c-myc: (sense) 5′-TGGTGCTCCATGAGGAG ACA-3′;
c-myc: (antisense) 5′-GTGTTTCAACTGTTCT CGTC-3′.
*β*-actin: (sense) 5′-GCACCACACCTTCTA CAATG-3′;
*β*-actin: (antisense) 5′-TGCTTGCTGATCCACATC TG-3′.
HK II: (sense) 5′-ACAATGGATGCCTAGATG-3′
HK II: (antisense) 5′-AGGTACATTCCACTG ATC-3′
PFKP: (sense) 5′-ACCACCGATGATTCCATT-3′
PFKP: (antisense) 5′-CTTGAGCCACCACTGT TC-3′
LDHA: (sense) 5′-TGGTTGAGAGTGCTTATG-3′
LDHA: (antisense) 5′-GCCTAAGATTCTTCAT TATACT-3′
PKM2: (sense) 5′-CCACTTGCAATTATTTGA GGAA-3′
PKM2: (antisense) 5′-GTGAGCAGACCTGCCAGACT-3′

### Analysis of cell proliferation

U2OS and S180 cells were planted in 96-well culture plates (5×10^3^ cells per well for U2OS and 1×10^4^ cells per well for S180). For the measurement of glucose-dependent proliferation, RPMI 1640 containing glucose or no-glucose RPMI 1640 (Life technologies, Carlsbad, USA) supplemented with 4.5 g/L galactose (Sigma, St Louis, MO) was used as previously reported [20]. After incubation over night, medium contained serial concentrations of IS were replaced into the plates for 12, 24, 36 and 48 h. Cell viability was assessed by MTT assay [14]. To count the number of viable cells, Trypan Blue-negative cells were counted using a Countess Automated Cell Counter (Life technologies, Carlsbad, USA). In SW1353 cells, the incubation medium was replaced by DMEM medium and cell proliferation was assayed by the same method.

### EdU incorporation assay

According to the manual of a EdU labelling/detection kit (Ribobio, Guangzhou, China), U2OS and SW1353 cells were incubated in a final volume of 100 μl of complete medium at 1.25 × 10^4^ cells/well on 35-mm glass-bottom dish (Shengyou Biotechnology, Hangzhou, China). Following incubation overnight, the medium was replaced with fresh medium containing IS at different concentrations (20 - 30 μM) or DMSO as vehicle (< 0.1 %). 24 hrs later, 50 μM EdU labeling agent was added to the cell culture and incubated for another 8 hrs at 37°C under 5 % CO_2_. The cells were fixed with 4% paraformaldehyde (pH 7.4) for 30 min and incubated with glycine for 5 min. Tthe cells were washed with PBS and stained with anti-EdU working solution at room temperature for 30 min. The cells were washed again with 0.5% TritonX-100 in PBS and incubated with 5 μg/ml Hoechst 33342 at room temperature for 30 min. The cells were observed under a confocal laser scanning microscope LSM 700 (Carl Zeiss, Oberkochen, Germany). The number of EdU-positive cells was calculated from five random fields in three wells.

2 × 10^6^ S180 cells were incubated with 10 ml fresh medium containing IS at different concentrations (20 - 30 μM) or DMSO as vehicle (< 0.1 %). 24 h later, 50 μM EdU labeling agent was added to the cell culture to incubate for another 8 hrs at 37°C under 5 % CO_2_. Cells were fixed with 4% paraformaldehyde (pH 7.4) for 30 min, washed with PBS and stained with anti-EdU working solution at room temperature for 30 min. Following washed with PBS, the cells were analyzed by flow cytometry (488 nm excitation and 525 nm emission filters) using BD Accuri™ C6 flow cytometry (Becton & Dickinson Company, Franklin Lakes, NJ).

### Sarcoma xenograft mouse model

The mouse-xenograft model was established by subcutaneous injection of 2 × 10^6^ S180 cells into the right armpit of 5-week old ICR male mice. The mice were randomized into 5 groups (8 - 10 mice per group): saline control group, 30 mg/kg 5-FU, 10, 20, 30 mg/kg IS group when xenografts were palpable. Vehicle or drugs were administered intravenously everyday; body weight was measured and recorded every day. On day 9, mice were euthanized killed; tumors were collected, weighed, and photographed. The tumor inhibition effect of IS on tumor growth was calculated using the following equation: tumor suppression (%) = (1-T/C) × 100 %, where T is the average tumor weight of the treated group and C is that of the control group. The institutional and national guidelines for the care and use of animals were followed and the Ethical Committee of China Pharmaceutical University approved the current study.

### Histological examination

For histological analysis, tumors of all groups were obtained and fixed in 10 % neutral-buffered formaldehyde for 48 h, embedded in paraffin, and sliced at 5 μm thickness. The sections were stained with haematoxylin and eosin (H&E), and examined by light microscopy [14].

### Statistical analysis

All experiments were performed at least 3 times unless otherwise stated. The results were analyzed using one-way ANOVA with Tukey multiple comparison test. The data are given as the mean ± S.D.. *P* value less than 0.05 was considered as significant.

## SUPPLEMENTARY MATERIALS AND METHODS FIGURES



## Abbreviations

mTORC1/2: mammalian target of rapamycin complex 1/2
4E-BP1: eukaryotic translation initiation factor 4E-binding protein 1
IS: Icariside II
5-FU: 5-fluorouracil
mLST8: mammalian lethal with SEC13 protein 8
PRAS40: proline-rich Akt substrate 40
Ser: Serine
Thr: Threonine
