# Supplementary material for: Icariside II, a natural mTOR inhibitor, disrupts aberrant energy homeostasis via suppressing mTORC1-4E-BP1 axis in sarcoma cells
Source: Oncotarget. 2016 Apr 1;7(19):27819–37. doi: 10.18632/oncotarget.8538 (PMC5053690; doi:10.18632/oncotarget.8538)
Supplement: Supplementary file 1 [file oncotarget-07-27819-s001.pdf]

SUPPLEMENTARY MATERIALS AND METHODS

Materials

IS (purity > 99%) was dissolved by dimethyl sulfoxide (DMSO). The DMSO concentration in all drug-treated cells was less than 0.1 %. MTT and 5-FU were purchased from Sigma (St Louis, MO).

Cell culture

Human normal osteoblast hFOB 1.19 cells were purchased from Cell Bank of Shanghai Institute of Biochemistry and Cell Biology, Chinese Academy of Sciences (Shanghai, China). hFOB 1.19 cells were cultured in DMEM/F12 media supplemented with 0.3 mg/mL G418, 10% fetal bovine serum and incubated at 33.5°C with 5 % CO_2.

Analysis of cell proliferation

hFOB 1.19 cells were planted in 96-well culture plates (1×10^4 cells per well for hFOB 1.19). After incubation over night, medium contained serial concentrations of IS were replaced into the plates for 12, 24, 36 and 48 h. To count the number of viable cells, Trypan Blue-negative cells were counted using a Countess Automated Cell Counter (Life technologies, Carlsbad, USA).

Body weight and histological analysis

The mouse-xenograft model was established by subcutaneous injection of 2×10^6 S180 cells into the right armpit of 5-week old ICR male mice (National Rodent Laboratory Animal Resource, Shanghai, China). The mice were randomized into 3 groups (6-8 mice per group): saline control group, 30 mg/kg 5-FU and 30 mg/kg IS group when xenografts were palpable. Vehicle or drugs were administered intravenously everyday; body weight was measured and recorded every day. On day 9, mice were killed. Organs were collected and fixed in 10% neutral-buffered formaldehyde for 48 h, embedded in paraffin, and sliced at 5 μm thickness. The sections were stained with haematoxylin and eosin (H&;E), and examined by light microscopy. The institutional and national guidelines for the care and use of animals were followed and the Ethical Committee of China Pharmaceutical University approved the current study.

Statistical analysis

All experiments were performed at least 3 times unless otherwise stated. The results were analyzed using one-way ANOVA with Tukey multiple comparison test. The data are given as the mean ± S.D.. *P* value less than 0.05 was considered as significant.

SUPPLEMENTARY FIGURES

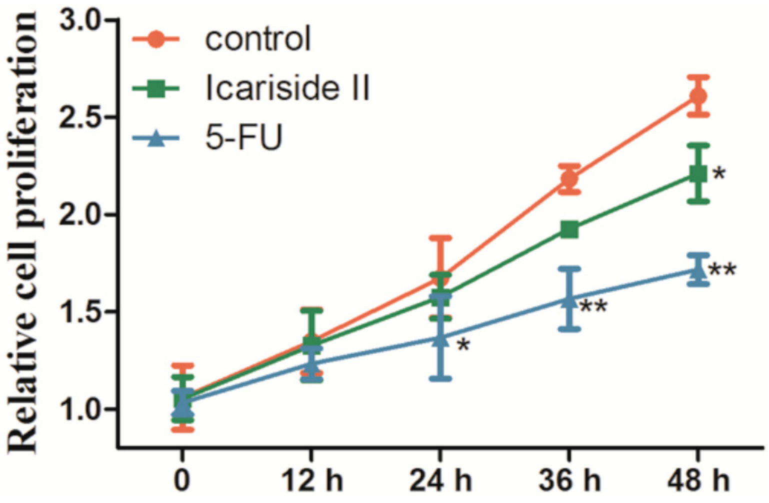

Supplementary Figure S1: Growth curves of IS or 5-FU treated normal osteoblast hFOB 1.19 cells. *p < 0.05 and **p < 0.01 versus control group.

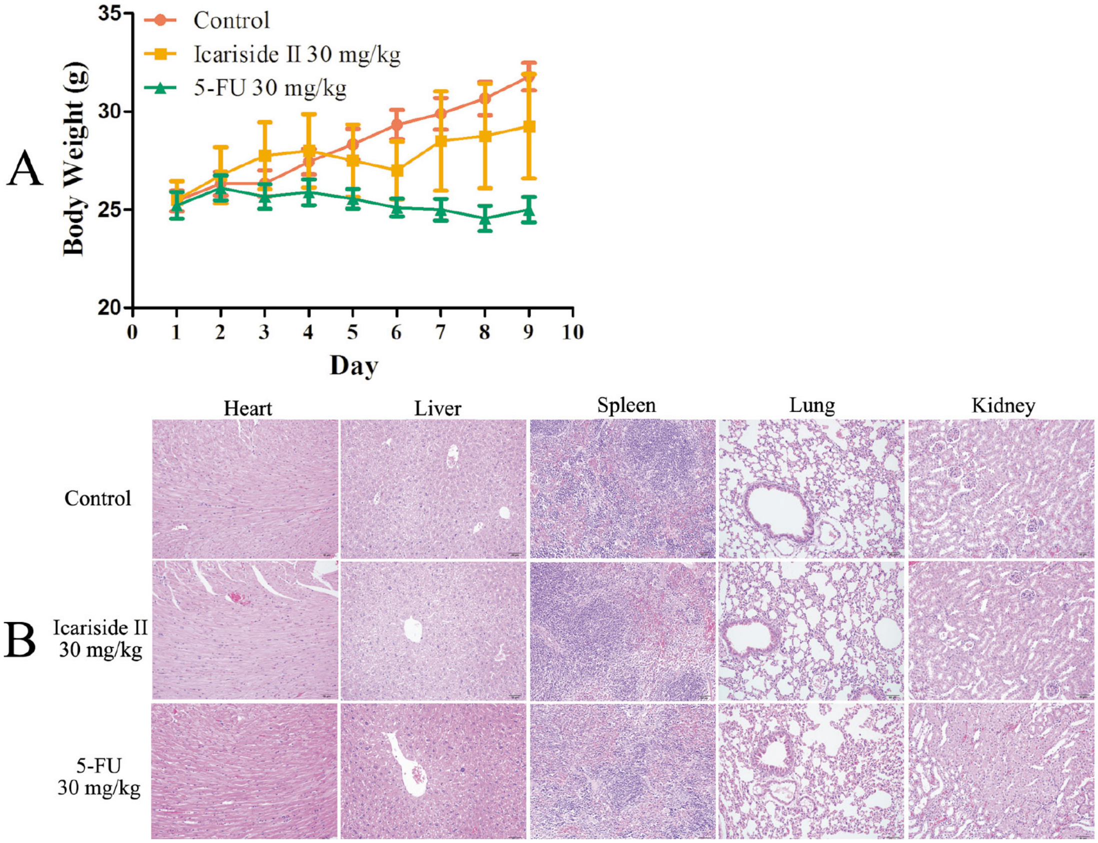

Supplementary Figure S2: Body weight and histological analysis. A. The average weight of treated and control mice was recorded every day. **B.** The organs from treated and control groups were stained with H&;E to evaluate the side effect of IS.
